# Involvement of Androgen Receptor in Sex Determination in an Amphibian Species

**DOI:** 10.1371/journal.pone.0093655

**Published:** 2014-05-14

**Authors:** Jun Fujii, Maho Kodama, Akira Oike, Yasuki Matsuo, Mi-Sook Min, Takashi Hasebe, Atsuko Ishizuya-Oka, Koichi Kawakami, Masahisa Nakamura

**Affiliations:** 1 Department of Biology, Waseda University, Tokyo, Japan; 2 Laboratory of Wildlife Conservation Genetics, Seoul National University, Seoul, South Korea; 3 Department of Biology, Nippon Medical School, Kawasaki, Kanagawa, Japan; 4 Division of Molecular and Developmental Biology, National Institute of Genetics, Mishima, Shizuoka, Japan; University of Hyderabad, India

## Abstract

In mice and humans, the androgen receptor (*AR*) gene, located on the X chromosome, is not known to be involved in sex determination. In the Japanese frog *Rana rugosa* the *AR* is located on the sex chromosomes (X, Y, Z and W). Phylogenetic analysis shows that the *AR* on the X chromosome (*X-AR*) of the Korean *R. rugosa* is basal and segregates into two clusters: one containing *W-AR* of Japanese *R. rugosa*, the other containing *Y*-*AR*. *AR* expression is twice as high in ZZ (male) compared to ZW (female) embryos in which the *W*-*AR* is barely expressed. Higher *AR*-expression may be associated with male sex determination in this species. To examine whether the *Z*-*AR* is involved in sex determination in *R. rugosa*, we produced transgenic (Tg) frogs carrying an exogenous *Z*-*AR*. Analysis of ZW Tg frogs revealed development of masculinized gonads or ‘ovotestes’. Expression of *CYP17* and *Dmrt1*, genes known to be activated during normal male gonadal development, were up-regulated in the ZW ovotestis. Testosterone, supplied to the rearing water, completed the female-to-male sex-reversal in the *AR*-Tg ZW frogs. Here we report that *Z*-*AR* is involved in male sex-determination in an amphibian species.

## Introduction

**Figure 1:**
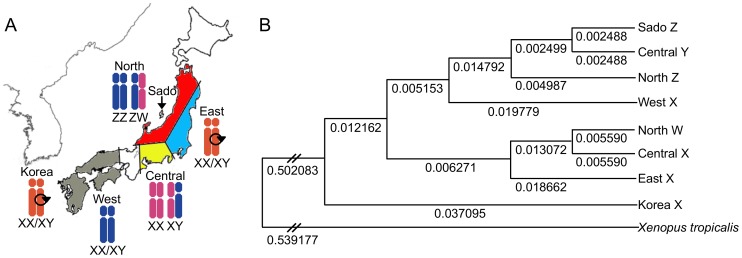
Six geographic populations and phylogenetic tree. (A) Six geographic populations differing in their morphology of the sex-determining chromosome. This figure, modified from Ref. [4]–[6], shows five local populations in Japan and one in Korea. Three local populations in Japan and one population in Korea have the XX/XY sex-determining system, whereas two populations have the ZZ/ZW. The four geographic populations in Japan are shown in different colors. Sado Island is located in northern Japan as indicated by a black arrow. The Z of northern, and X and Y of western Japan are subtelocentric. The W of northern and X of central Japan are metacentric, and the X and Y of Korea and eastern Japan are more subtelocentric. Pericentric inversions are indicated by a circle with an arrowhead. (B) Phylogenetic tree of the *AR* gene from different populations of *R. rugosa*. The tree was constructed by the UPGMA method, using *Xenopus tropicalis AR* (NM001090884) as an outgroup. The Korean *R. rugosa AR* (AB910584) is basal. All other *AR*s segregate into two main clusters: the X of western (AB910586), Y of central (AB910589) and Z of northern and Sado island *R. rugosa* (AB910592); and the X of eastern (AB910585) and central (AB910588), and the W of northern Japan (AB910591).

Sex is genetically determined in most vertebrates. As in other vertebrate species, heterogametic sex chromosomes in amphibians determine the male (XX/XY) or female (ZZ/ZW) fate [1], [2]. The Japanese frog *R. rugosa* (2n = 26) has two sex-determining systems within one species [3]. Frogs living in eastern, western and central Japan have the XY system, whereas those in northern Japan have the ZW system (Fig. 1A). Furthermore, frogs living in northern and central Japan have heteromorphic sex chromosomes, whereas those living in eastern and western Japan are homomorphic (Fig. 1A). The ancestral or basal-type sex chromosomes of the Japanese *R*. *rugosa* are found in the Korean *R. rugosa* [4]. It has been proposed that the XY and ZW sex chromosomes of *R. rugosa* evolved through two independent inversions on chromosome 7 [5], [6]. However, a sex-determining gene has not yet been found in *R. rugosa*.

Androgens exert a variety of effects in target tissues such as male reproductive organs, brain and skeletal tissues. Androgenic effects are mediated by tissue-specific transcriptional control of target genes via nuclear androgen receptor (AR) [7]. In *AR*-knockout mice [8], [9], males have a female-like appearance and body weight, but the sex remains male; the testes become smaller but spermatogenesis is observed, although arrested predominantly at the diplotene stage of premeiosis. Female-to-male sex-reversal is not observed in *AR* knock-in mice [10]. Seemingly, therefore, the *AR* does not participate in sex determination in mice. The *Sry* on the Y chromosome is recognized as the master genetic determinant of male fate in this species [11].

In *R. rugosa* the *AR* gene is located on the sex (X, Y, Z and W) chromosomes and reportedly on the inverted region of the Y and W chromosomes [12], [13]. Structural rearrangements such as inversions, deletions and translocations are known to induce degradation of native genes by accumulation of deleterious mutations [14]. Thus, it is likely that the *AR* gene is in the process of evolutional degradation from lack of recombination between the inverted and non-inverted regions of the sex chromosomes (X vs. Y, and Z vs. W). In addition, it has been proposed that degradation of the *W-AR* began just after or at the origin of the ZW sex-determining system in *R. rugosa* [15]. In fact, we previously reported that *W*-*AR* expression levels are extremely low in *R. rugosa* embryos, perhaps owing to promoter sequence variation and cognate transcription factor interaction between the *W*- and *Z-AR* promoters [16]. However, W-AR proteins can trans-activate androgen-dependent transcription when *W*-*AR* is transgenically expressed as demonstrated in reporter assays using *Xenopus laevis* kidney-derived A6 cells [16]. Thus, degradation of *R. rugosa W-AR* is still in an early phase. During sex determination, *R. rugosa* male gonads synthesize more androgens than females [17]. In addition, *AR* expression is up-regulated in the male gonad of *R. rugosa* tadpoles prior to sex determination [16]. These findings led us to speculate that the *AR* may be involved in male sex determination in this species. To test this hypothesis, we produced transgenic (Tg) frogs carrying an exogenous *Z*-*AR* driven by the promoter region of both *Z-AR* and *EF1α* genes. Strikingly, a subset of the *Z-AR*-Tg female (ZW) frogs formed composite gonads or “ovotestes”. Here we report that the *Z*-*AR* is involved in male sex determination in a vertebrate species.

## Materials and Methods

### Ethics Statement

All the animal experiments in this study were performed in respect of the Fundamental Guidelines for Proper Conduct of Animal Experiment and Related Activities in Academic Research Institutions (Notice No. 71 of the Ministry of Education, Science, and Culture of Japan, 2006) and the Prevention of Cruelty to Animal Act (Notice No. 88 of the Ministry of the Environment of Japan, 2006). This included official approval from the Committee of Animal Experimentation of Waseda University (Permit Number: 2013-A005).

### Animals

Female heterogametic (ZZ/ZW) *R. rugosa* frogs were used in this study. Unfertilized eggs were artificially ovulated and inseminated [16]. Fertilized eggs were dejellied with 2% (w/v) cysteine in 0.1×MMR (1×MMR: 100 mM NaCl, 2 mM KCl, 1 mM MgSO4, 2 mM CaCl2, 5 mM HEPES, pH 7.6). The eggs were developed to tadpoles at various developmental stages and to frogs just after metamorphosis; they were staged by criteria described elsewhere [16]. The genetic sex of tadpoles was determined as reported previously [16].

### Construction

#### Phylogenetic tree of the AR gene

To construct a phylogenetic tree of the AR gene, we employed PCR to amplify the promoter region of the *AR* using primers shown in Table 1. The PCR reaction consisted of 4 min at 94°C, followed by 35 cycles of 95°C (30 sec), 65°C (30 sec), and 72°C (1 min), ending with 7 min of extension at 72°C. The nucleotide sequence of amplified DNA was determined as described previously [16]. An *AR* gene tree was constructed using the UPGMA method [Genetyx Mac version 14.0.9].

**Table 1:** Primers used for PCR analysis.

Integration of transgene and transgene expression		
*Z-AR/V5*	F	5′-GCGGTTTTTCCAACTTACCA-3′
	R	5′-CGAGACCGAGGAGAGGGTTA-3′
*Z-AR*	F	5'-CCGATGAAGACCGAGATGAACC-3'
	R	5'-CTGCGGCGGGAAGTAATAGTC-3'
*CYP17A1*	F	5′-CGCTGTGTATGTTCGGTGAAGG-3′
	R	5′-GGTCTCGAGCTGCCACTGACT-3′
*CYP19*	F	5′-ACATTGGCCGCATGCATAAA-3′
	R	5′-CGGGGCTGTGTGCAGAGAAA-3′
*Dmrt1*	F	5′-GCCTGTGTTCCACCATTCTT-3′
	R	5′-GCCACGCAGATCATAAAATTG-3′
*GA3PDH*	F	5′-GAAGTGAAGGCTGACGGAGGA-3′
	R	5′-CGCCTTGTCATAGCTTTCATGGT-3′
Genomic DNA manipulation		
*AR* of eastern X, western X, northern Z, Sado Z and central Y		
1^st^ PCR	F	5′-CTTCACAACATGTCGCTCGT-3′
	R	5′-GGCTTTGCCAGCAGAATAAG-3′
2^nd^ PCR	F	5′-ATTTCTCTCCTCCTGCGTGA-3′
	R	5′-TCTTGTCCTCCTCAGCCAACT-3′
*AR* of Korean X, northern W and central X		
1^st^ PCR	F	5′-CATAAAGGCTGCCCATCAGT-3′
	R	5′-CATAAAGGCTGCCCATCAGT-3′
2^nd^ PCR	F	5′-CAATCAGTGCCGCATATCAC-3′
	R	5′-CTAAAGGCACCCTCACGGTA-3′

F:Forward primer, R:Reverse primer.

#### AR-expression vector

**Figure 2:**
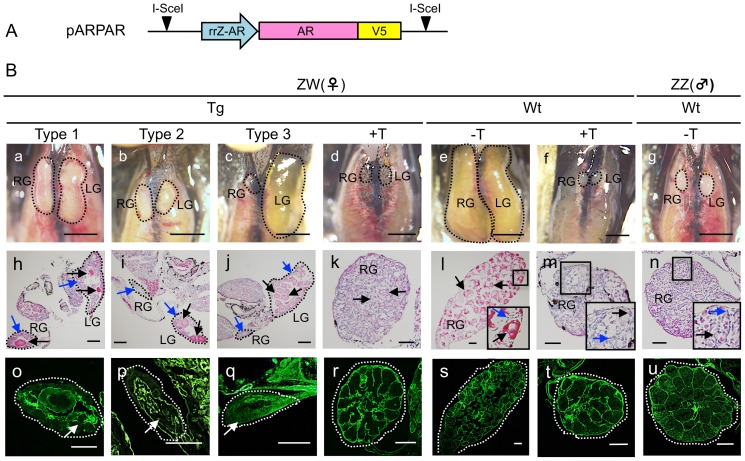
Histology of gonads. (A) Schematic diagram of the Tg vector, pARPAR, 1 of 2 Tg vectors used in this study. (B) Histology of Tg and Wt gonads. Tg and Wt gonads with (+) or without (−) T-treatment were taken after laparotomy (upper figures, a–g; bar = 2 mm). Sections from Wt and Tg right and left gonads (RG and LG) were stained with Hematoxylin & Eosin (middle figures, h–n; bar = 100 µm) and a laminin antibody (lower figures, o–u; bar = 100 µm). Dashed lines indicate the borders of the gonads. In the middle figures, germ and somatic cells are indicated by black and blue arrows, respectively. Magnified images of the area within the square are shown in (l), (m) and (n).

To construct the vectors for transgenesis we inserted the 2.3-kbp *Z-AR* cDNA (pink bar) and the promoter of *Z-AR* (blue arrow) into the p(I-SceI)DPCG construct (pARPAR, Fig. 2A) [18], or the *R. rugosa EF1α* (blue arrow) into the pT2AL200R150G (prrT2ARG). First, we amplified the *Z-AR* cDNA by PCR using a pair of primers specific for the nucleotide sequence of *Z-AR* cDNA: forward 5′-AAAAAAGATATCATGGAGGTGCACATTGGACT-3′ and reverse 5′-GAGATGAAGGTGTCAAGTACCTATAGAAAAAA-3′, and the *R. rugosa Z*-*AR* (DDBJ Accession No. AB491761) or *EF1α* (AB822986) promoter region. Then we inserted the *Z-AR* (−1,359 to −1) or *EF1α* (−1,695 to −1) promoter region ligated to the 2,312-bp *Z-AR* cDNA into the p(*I-Sce*I)DPCG vector between the *Sal*I/*Age*l sites and the pT2AL200R150G vector [18] between the *Sal*I/*Xho*I sites, respectively. Each *AR* expression vector carried either the promoter of the *AR* gene or of *EF1α*. V5 (yellow bar) represents the small epitope tag polypeptides (GKPIPNPLLGLDST) of the V5 protein from the paramyxovirus of simian virus 5 (SV5). Restriction enzyme sites for *I-Sec*I and *Tol*II recognition sites are indicated by black arrowheads.

### *AR*-Transgenesis

#### Production of Tg frogs

To produce Tg frogs, we used the *I-Sce*I meganuclease- [18] and transposon-mediated gene trap [19]. Fertilized eggs were injected with *I-Sce*I meganuclease (NEB) and the *I-Sce*I-cleaved plasmid encoding Z-AR and V5, and the *Tol* II mRNA [19] and the prrT2ARG plasmid encoding Z-AR and GFP, respectively, using a NANOJECT II injection apparatus (Drummond). Tg embryos were cultured in 0.1×MMR with 6% Ficoll PM400 (GE Healthcare) and 50 µg/ml gentamicin (Wako), developed to St. 20 at 18°C, and then transferred to water at room temperature. The tadpoles were continuously reared in water with or without T (50 ng/ml; 150 nM). To confirm the exogenous *Z-AR* integration into genomic DNA, we extracted DNA from the tail tip of all Tg frogs just after metamorphosis, using the AllPrep DNA/RNA Micro Kit (QIAGEN). The PCR primers used were: forward, 5′-GCGGTTTTTCCAACTTACCA-3′ and reverse, 5′-CGAGACCGAGGAGAGGGTTA-3′).

#### Transgene expression in gonads

We employed PCR analysis to examine *Z-AR/V5* expression in Tg ZW gonads. Total RNA was prepared from the gonads of Wt and Tg frogs just after metamorphosis using ISOGEN (NIPPON GENE) and cDNAs were synthesized [16]. The PCR reaction consisted of 4 min at 94°C, followed by 35 (*Z-AR*/*V5*, *CYP17A1*, and *Dmrt1*) or 25 cycles (*GA3PDH*) of 95°C (30 sec), 62°C (30 sec), and 72°C (1 min), ending with 7 min of extension at 72°C. DNA fragments for *Z-AR*/*V5* (260-bp), *CYP17A1* (330 bp, AB284119), *Dmrt1* (374-bp, AB272609) and *GA3PDH* (252-bp, AB284116) cDNAs were amplified by PCR using a set of primers for each respective template. Primer sequences are given in Table 1.

### Immunohistology

#### Production of anti- AR and anti-CYP17 antibodies

For production of mouse antibodies, we designed two sets of primers. One pair amplified the *Z-AR* cDNA (AB372103) encoding polypeptide RVISCKRNNPASSSRRFFQL (AR20) corresponding to residues 695–714, and the other the *CYP17A1* cDNA (AB284119) encoding polypeptide DEKEWVNPHLFNPDRFLDENGNRVYS (CYP1726), corresponding to residues 409–434. The DNA fragments were inserted into the *Bam*HI/*Xho*I sites of the pGEX-4T expression vector (GE Healthcare). The GST-AR20 and GST-CYP1726 expression cassettes were transfected into *E*. *coli* BL21. The transfected cells were sonicated in ice-cold water using an Ultrasonic Processor (TAITEC, model VP-ST). We purified GST-AR20 and GST-CYP1726 proteins according to the manufacturer's protocol (GE Healthcare) and immunized 8-week-old BALB/c female mice three times with 50 µg of protein in complete Freund's adjuvant (Wako) at 2-week intervals. Sera were collected and tested 5 days after the last booster injection and used for immunohistochemistry.

#### Western blot analysis

*R. rugosa* adult testes protein (30 µg) and molecular weight markers (BIO-RAD) were subjected to electrophoresis using a 10% polyacrylamide gel and 30 mA for 1 to 2 hrs [20]. Proteins were transferred to Amersham Hybond-P (GE Healthcare) at 25 V for 1.5 h using a Semi Dry transfer instrument (BIO CRAFT; BE 310). Membranes were blocked for 2 h at RT in 5% skim milk (w/v; Wako)/TBS-TX (TBS+0.1% Triton X-100). The antibodies against AR and CYP17 were used at a 1∶1,000 dilution followed by anti-mouse goat IgG conjugated with horseradish peroxidase (Sigma-Aldrich) as a secondary antibody. Subsequently, the membrane was washed three times in TBS-TX for 10 min each time. The blot was then incubated in ECL Plus Western Blotting Detection Reagent for 5 min at RT. Signals were detected using an LAS-300 imager (FUJIFILM). Before immunohistochemical analysis, we validated the specificity of the CYP17 and AR antibodies by immunoblotting using a homogenate of *R. rugosa* adult testes.

#### Immunostaining

The primary antibodies against Vasa [20] and CYP17 were prepared in our laboratory and the anti-laminin antibody was purchased from Sigma-Aldrich.

Gonads were surgically excised from Wt and Tg frogs just after metamorphosis, fixed and frozen as previously described [21]. Frozen tissues were cut at 8-µm thickness with a Leica cryostat (Leica, CM1850) and placed on glass slides. The sections were incubated overnight at 4°C with antibodies at 1∶1,000 dilution. Following incubation with Alexa Fluor 488 goat anti-rabbit or anti-mouse secondary antibodies (Life Technologies), slides were counterstained with DAPI (4′, 6-diamidino-2-phenylindole; Life Technologies). Fluorescent signals were detected under fluorescence (OLYMPUS, model BX51) and confocal (OLYMPUS, model FV1000) microscopy. Sections were also counter-stained with Hematoxylin & Eosin and subjected to histological observations under a light microscope (OLYMPUS, model BX51).

### Image acquisition and analysis

Images were scanned and adjusted for brightness and contrast by Adobe Photoshop CS2.

## Results

### Phylogeny of the *AR* gene

An ancestral type of the sex chromosomes of *R. rugosa* is found in the Korean *R. rugosa* [4]. To examine whether the *W-AR* gene was inherited from the ancestral type *X*-*AR* of the Korean *R. rugosa*, we constructed an *AR* gene tree using the UPGMA method [Genetyx Mac version 14.0.9], based on the nucleotide sequences of the promoter region of the *AR* in the 6 local populations. The *X*-*AR* in the Korean population appeared to be basal and segregated into two clusters: one contained the *X-AR* of western Japan, the *Z-AR* northern Japan including Sado island and the *Y-AR* of the central population; the other contained the *X-AR* of eastern and central Japan and the *W-AR* of the northern population (Fig. 1B). The clusters coincided well with the morphology chromosome 7 of the sex chromosomes [5].

### Histology of transgenic gonads

When Tg ZW embryos carrying an exogenous *Z*-*AR* driven by the *Z-AR* promoter (pARPAR, Fig. 2A) were developed into frogs just after metamorphosis, a subset of the Tg frogs developed masculinized gonads (Figs. 2Ba–c) in place of normal ovaries (Fig. 2Be). Based on macroscopic observations we classified the Tg gonads into three categories: Type 1, both right (RG) and left gonads (LG) were approximately 50% smaller than the normal ovary and containing many somatic cells (dark cells, blue arrows) and fewer oocytes (red cells, black arrows) (Figs. 2Ba and h); Type 2, both gonads were 25% the size of normal ovaries with fewer oocytes (black arrows) and many somatic cells (blue arrows) (Figs. 2Bb and i); Type 3, the RG appeared macroscopically as a testis without any oocytes, while the LG was a normal ovary with many oocytes (black arrows) as well as somatic cells (blue arrows) (Figs. 2Bc and j). We observed 3 frogs carrying the type 3 Tg gonads showing lateral heterogeneity with the R a testis-like gonad whereas the LG was a normal ovary (Fig. S1). The results collectively indicate that *AR*-transgenesis is associated with female-to-male sex-reversal in *R. rugosa*, although this reversal is incomplete in comparison to wild-type (Wt) testis. Wt female (ZW) and male (ZZ) frogs developed normal bilateral ovaries and testes, respectively (Figs. 2Be and g). Many oocytes (black arrows) and somatic cells (blue arrow) were observed in the ZW ovary (Fig. 2Bl, insert), and many germ cells (black arrow) and somatic cells (blue arrow) were observed in the ZZ testis (Fig. 2Bn, insert).

Tg ZW frogs carrying the exogenous *AR* developed testes like those in ZZ frogs when reared in water containing testosterone (T) (50 ng/ml; 145 nM) (Figs. 2Bk and n). Many germ cells were observed as indicated by black arrows (Fig. 2Bk). Similarly, Wt ZW frogs developed testes when ZW tadpoles were reared in water containing T (Fig. 2Bm).

To examine the internal structure of non-Tg and Tg gonads, tissue sections were immunostained for laminin, a marker of the basement membrane [21]. Laminin staining showed that the basement membranes surrounded many oocytes in the ovary (Fig. 2Bs) and the seminiferous tubules in the testis (Fig. 2Bu). However, the membranes became disrupted during sex-reversal. In the Type 1, 2 and 3 Tg gonads, basement membranes surrounding the oocytes in the ovary become disrupted during sex-reversal, an indication that the process of sex-reversal had been initiated (Figs. 2Bo, p and q; white arrow). Basement membranes were, however, found to surround the seminiferous tubules in the Tg and Wt T-induced ZW testes (Figs. 2Br and t), contrasting with the laminin structure of the female ZW ovary (Fig. 2Bs). The seminiferous tubules were organized in the Tg ZW testis (Fig. 2Br) than in the Wt ZW testis (Fig. 2Bt).

### Analysis of transgenic frogs

We introduced an exogenous *AR* driven by the *Z-AR* promoter (pARPAR) into 450 fertilized eggs of *R. rugosa*. Two hundred and thirty injected eggs (51.1%) developed into tadpoles at St. I (limb buds are visible) and 222 tadpoles (49.3%) grew into frogs just after metamorphosis, of which 111 were characterized (Table 2). In the absence of T in the rearing water, 76 tadpoles metamorphosed into frogs. As expected, all 30 non-Tg male (ZZ) frogs developed testes and all 27 non-Tg (Wt) ZW tadpoles developed into female frogs with ovaries. However, 8 out of 19 *AR*-Tg ZW female frogs (42.1%) developed an intermediary gonad, a hybrid of testis and ovary, called ‘ovotestis’. These hybrid organs were classified into the three categories mentioned above (Types 1, 2 and 3).

**Table 2:** Genotype and phenotype of transgenic frogs.

Vector	Karyotype	Transgene	Testosterone	Frogs(No.)	Ovotestis			testis	ovary
					Type 1	Type 2	Type 3		
pARPAR	ZW	+	-	19	3	2	3	-	11
	ZW	-	-	27	-	-	-	-	27
	ZZ	-	-	30	-	-	-	30	-
	ZW	+	+	9	-	-	-	9	-
	ZW	-	+	12	-	-	-	12	-
	ZZ	-	+	14	-	-	-	14	-
				111	3	2	3	65	38
prrT2ARG	ZW	+	-	7	1	-	-	-	6
	ZW	-	-	1	-	-	-	-	1
	ZZ	+	-	6	-	-	-	6	-
	ZZ	-	-	1	-	-	-	1	-
				15	1	-	-	7	7

When T was present in the rearing water, 35 tadpoles metamorphosed into frogs. All 14 of the Wt male (ZZ) frogs developed testes, as expected. All 12 of the Wt ZW frogs and all 9 of the *AR*-Tg ZW frogs formed testes.

In an alternative transgenic strategy, when we injected an exogenous *AR* driven by the *R. rugosa EF1α* promoter (prrT2ARG) into 200 fertilized eggs (Table 2), 76 (38.0%) developed into tadpoles at St. I, 71 (93.4%) of which grew into frogs just after metamorphosis. Of 15 Tg frogs analyzed just after metamorphosis 7 were male (ZZ) and 8 female (ZW). One out of the 7 Tg female (ZW) frogs formed ovotestes Type 1 (Table 2), thus confirming that *AR* transgenesis initiates sex-reversal in female *R. rugosa* frogs. All ZZ males developed testes.

### Expression of the *Z-AR* transgene

**Figure 3:**
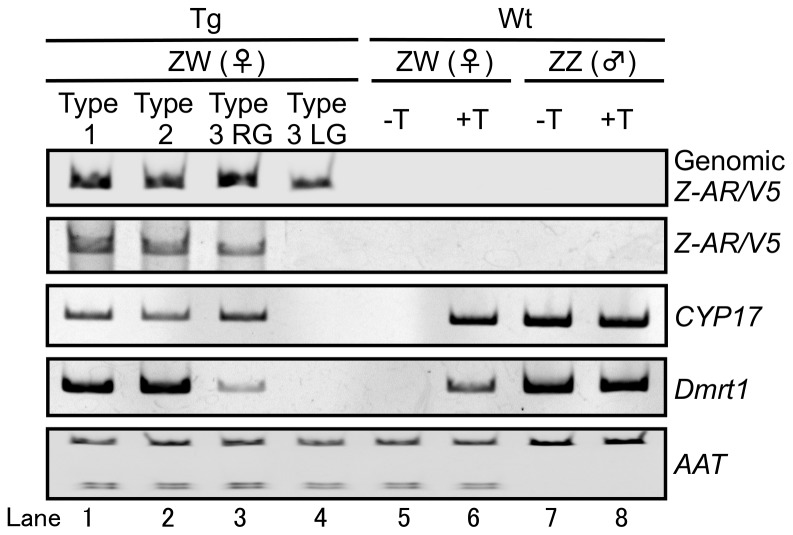
*Z-AR/V5*, *CYP17* and *Dmrt1* mRNA expression and *Z-AR/V5* and *AAT* genomic analysis. Genomic DNA PCR amplification was performed for the transgene (genomic *Z-AR/V5*). The sex of each frog was determined by genomic amplification of the ATP/ADP translocase (*AAT*) gene as previously described [16]. RT-PCR analysis was used to detect *Z-AR/V5*, *CYP17* and *Dmrt1* mRNA in Wt and Tg gonads treated with (+) or without (−) T. Top panel, *Z-AR*/*V5* integration into genomic DNA; 2nd panel, *Z-AR*/*V5* expression; 3rd panel, *CYP17* expression; 4th panel, *Dmrt1* expression; bottom panel, *AAT* genetic sex determination of each frog.

We confirmed the genomic integration of the *Z*-*AR/V5* gene driven by the promoter region of the *Z-AR* by PCR analysis in all the Tg ZW *R. rugosa* individuals tested (Fig. 3, top panel). Gene expression analysis revealed that the *AR*/*V5* mRNA was transcribed in both Type 1 and Type 2 Tg gonads, as well as in the Tg right gonad (RG) of Type 3, but not the Tg left gonad (LG, i.e. similar to the Wt ZW ovary; Figs. 2c and e) (Fig. 3). No transcripts for *AR*/*V5* were detectable in either Wt ZW ovary or Wt ZZ testis (Fig. 3, lanes 5 & 6 and 7 & 8, respectively). In addition, the Tg-ZW frogs not showing signs of masculinization in the gonads also expressed the *AR*. The Type 3 LG showed a little less expression of *AR* compared to that of the Type 3 RG (Fig. S2). However, we could not determine whether the difference in *AR* expression between the RG and LG was statistically significant, since the size of the RG tissues was too small for the requisite examination.

Previously we have shown that *CYP17* [22] and *Dmrt1* [23], [24] expression is increased in the XX sex-reversed gonad of *R. rugosa*. *CYP17* encodes an enzyme responsible for the conversion of progesterone into androstenedione, while *Dmrt1* is one of the genes controlling testicular differentiation in vertebrates [25]. When we examined the expression of these genes in the Tg ZW gonads, we found that *CYP17* and *Dmrt1* levels were elevated in both Type 1 and Type 2 masculinized Tg ZW gonads, as well as the Type 3 masculinized Tg RG but not in the non-masculinized Tg LG (Fig. 3, lanes 1–4). In the absence of exogenous T, the Wt ZW ovary did not transcribe detectable amounts of either *CYP17* or *Dmrt1* mRNA (Fig. 3, lane 5). However, the Wt ZW gonad did express both *CYP17* and *Dmrt1* following exposure to levels of T that induce sex-reversal (Fig. 3, lane 6). As expected, in the Wt ZZ testis, *CYP17* and *Dmrt1* were expressed regardless of T-treatment (Fig. 3, lanes 7 & 8). Therefore, exogenous *AR* can induce, in the genetically ZW female gonads, a process that involves the up-regulation of genes known to be responsible for hormone conversion and testis formation during normal gonadal development in Wt ZZ male frogs.

### Antibody producton and Western blot analyses

**Figure 4:**
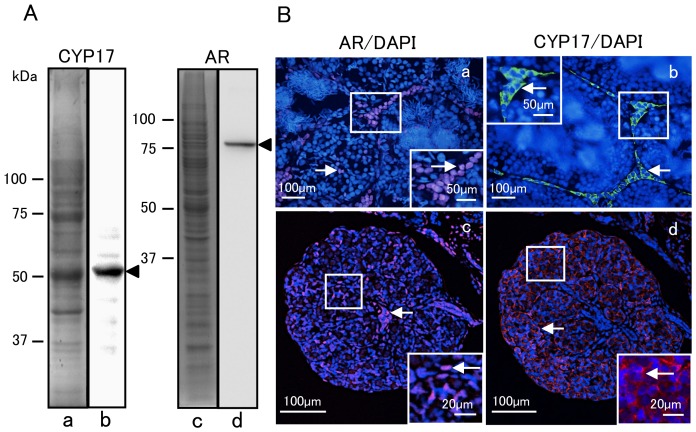
Western blot and immunohistochemical analyses. (A) Immunoblot analysis of adult testis homogenates with CYP17 and AR antibodies Black arrowheads indicate a single dominant band corresponding to CYP17 and AR with molecular weights of 56 (b) and 86 kDa (d), respectively. Panels (a and c) show the distribution pattern of testicular proteins that were electrophoresed and stained with Coomassie Blue. (B) Localization of AR and CYP17 in the testes from adult (a, b) and juvenile (c, d) frogs. Frozen sections from adult and juvenile testes were stained for AR (a, c) and CYP17 (b, d), and counter-stained with DAPI, respectively. White arrows indicate AR-positive signals in the nuclei of many interstitial cells and some germ cells in (a, c), while CYP17-positive signals in the cytoplasm of many interstitial cells are also indicated by white arrows (b, d). Magnified images of the area within the solid square are shown in (a–d).

We produced AR and CYP17 antibodies for detection of AR and CYP17 proteins expressed in the Tg gonad, and verified their specificity in Western blot analysis by using the homogenate of *R. rugosa* adult testes. The antibodies detected a single dominant band corresponding to CYP17 and AR with molecular weights of 56 (Fig. 4A-b) and 86 kDa (Fig. 4A-d), respectively, as indicated by black arrowheads. Panels (Figs. 4A-a and -c) show the distribution pattern of testicular proteins that were electrophoresed and stained with Coomassie Blue. To further demonstrate antibody specificity, frozen sections of adult testis were prepared for AR (Fig. 4B-a) and CYP17 (Fig. 4B-b) staining, and counter-staining with DAPI. The immunohistology showed that AR-positive signals were localized to the nuclei of many interstitial cells and some germ cells of adult (Fig. 4B-a) and juvenile (Fig. 4B-c) testes as indicated by white arrows, while CYP17-positive signals were produced in the cytoplasm of many interstitial cells of adult (Fig. 4B-b) and juvenile (Fig. 4B-d) testes. The results were confirmed by magnified images of the area within a solid square in Fig. 4B. Thus, we deemed these antibodies suitable for immunohistological studies.

### Immunohistology of *Z-AR* Tg gonads

**Figure 5:**
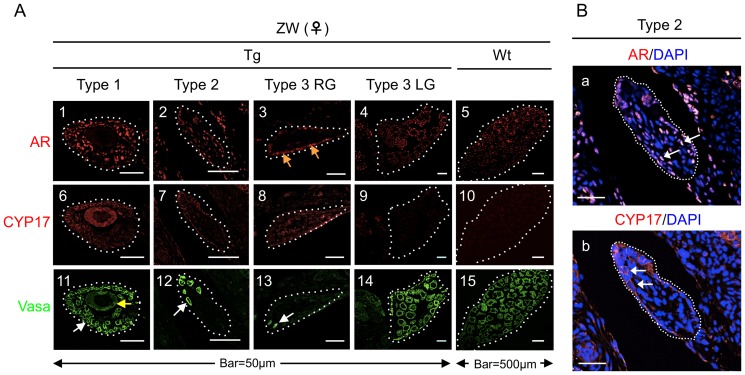
Immunohistology of ZW ovotestes. (A) Localization of AR, CYP17 and Vasa in the ovotestis frozen sections of Wt ZW ovary, and Type 1 to 3 ovotestes were stained for AR (1–5), CYP17 (6–10) and Vasa (11–15). A single oocyte and a small Vasa-positive cell are indicated by a yellow and white arrow, respectively. The orange arrows in (3) indicate AR-positive signals. (B) Localization of AR and CYP17 in the Type 2 ovotestis. Figures 2 and 7 in (A) are enlarged to (a) and (b) in (B), respectively. Frozen sections were stained immunohistologically for AR (a) and CYP17 (b), and counterstained with DAPI. Dashed lines indicate the borders of the gonads. AR- and CYP17-positive signals are indicated by white arrows in (a) and (b), respectively. Bar = 50 µm.

Tissue sections of non-Tg and Tg gonads were immunostained for CYP17, AR, and Vasa. Vasa is a germ cell marker [20]. The AR antibody used recognizes both endogenous and exogenous AR, since the amino acid sequences of both are identical. In Type 1 Tg ZW gonads, we observed few oocytes and many Vasa-positive germ cells (Fig. 5A11, yellow and white arrows, respectively). In the Type 2 category, several Vasa-positive germ cells were observed (Fig. 5A12, white arrow). AR expression was detected in the nuclei of somatic cells in the testis of Type 2 Tg ZW gonads (Figs. 5A2 and 5Ba, arrows), while CYP17 was detected in the cytoplasm of the somatic cells (Figs. 5A7 and 5Bb, arrows). For Type 3, in the RG, oocytes were entirely absent (Figs. 5A3), and very few small, Vasa-positive germ cells were observed (Fig. 5A13, white arrow). Additionally, some AR expression was observed in the peripheral region of this gonad, indicated by orange arrows (Fig. 5A3). In the Wt ZW ovary, many Vasa-positive oocytes were observed (Fig. 5A15), but no appreciable CYP17 expression was detected (Fig. 5A10). When Wt ZW female tadpoles were reared in water supplied with T, they formed testis expressing AR and CYP17 proteins (Fig. S3).

### Co-expression of AR and GFP in the ovotestes

**Figure 6:**
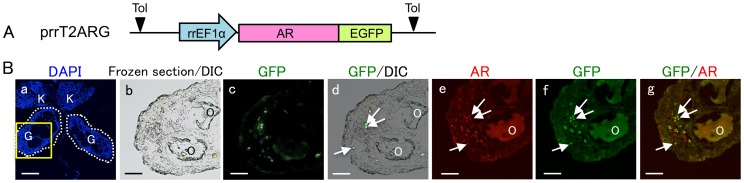
Co-expression of AR and GFP in the ovotestes. (A) Schematic diagram of Tg vector, prrT2ARG, 1 of 2 Tg vectors used in this study. (B) Frozen sections from the gonads of Z-AR/GFP Tg frogs just after metamorphosis were stained for AR and GFP and counterstained with DAPI. The letters G, K and O indicate gonad, kidney and oocyte, respectively. Dashed lines in (a) indicate the border of the gonads. The gonad in the boxed area with a yellow line in (a) was enlarged and examined under DIC (b, d) and fluorescence (c, d) microscopy. Some of the GFP-immuno-positive cells are indicated by white arrows (d, f, g). AR co-expression is indicated by white arrows in the same cells (e, g). Bar = 50 µm.

The Z-AR/GFP expression cassette driven by the promoter of the *R. rugosa EF1α* gene (prrT2ARG, Fig. 6A) was injected into fertilized eggs that were allowed to develop into frogs just after metamorphosis. Subsequently, 7 males (ZZ) and 8 females (ZW) were analyzed immunohistologically for AR and GFP expression. All males developed testes regardless of *Z-AR*/*GFP* transgenesis. However, 1 out of 7 Tg female (ZW) frogs formed ovotestes (Table 2). Differential Interference Contrast (DIC) and fluorescence microscopy revealed fewer oocytes (O) in the Tg ovotestis (Fig. 6B-b). Many GFP fluorescence signals were observed directly in the ovotestis sections (Figs. 6B-c and d), and further confirmed by indirect immunostaining with an anti-GFP antibody (Fig. 6B-f, white arrows). AR expression was also found in the ovotestis when sections were stained with the AR antibody (Fig. 6B-e), with AR and GFP co-localizing (Fig. 6B-g, white arrows), indicating that the *Z-AR*/*GFP* transgene was translated to produce the AR/GFP fusion protein.

## Discussion

In *R*. *rugosa*, the sex chromosomes of Korean frogs are basal [4]. Furthermore, the *Y*- and *W*-*AR* of the Japanese *R. rugosa* appear to have evolved from the basal type *X*-*AR* of the Korean *R. rugosa* (Fig. 1B). The *AR* is located on the inverted region of the Y and W chromosomes of Japanese *R. rugosa* [12], [13]. Expression levels of *W*-*AR* are extremely low in *R. rugosa* embryos compared with *Z-AR* [16]. However, there is no difference in *AR* expression levels between XX and YY embryos of Japanese *R. rugosa* [15]. Therefore, it may be concluded that *W*- and *Y-AR* of Japanese *R. rugosa* evolved independently from the basal *X-AR* of Korean *R. rugosa*, and that *W-AR* of Japanese *R. rugosa* degraded more rapidly than *X*-, *Y*-, or *Z*-*AR* during sex chromosome evolution. Based on these findings, we hypothesized that *Z*-*AR* came to play a role, perhaps a critical one given its sex hormone-signaling function, in male sex determination in ZZ/ZW *R. rugosa*.

*X-AR* is not required for sex determination in mice and humans [8]–[10]. However in *R. rugosa* this fact remains untested. We reasoned that it would be possible to examine whether *Z-AR* is a critical gene for male sex determination in ZZ/ZW *R. rugosa* by analyzing female-to-male sex-reversal following induction of exogenous *Z-AR* into ZW embryos. Strikingly, a number of the *AR*-Tg ZW frogs developed varying degrees of masculinized gonad, which we have called ‘ovotestes’. The same phenomenon was reproducibly observed using another construct, a *Z-AR*/*GFP* expression vector driven by the 5′-flanking region of the *R. rugosa EF1α*, introduced into *R. rugosa* fertilized eggs. A female (ZW) frog formed ovotestes co-expressing AR and GFP. Hence, it is reasonable to conclude that *Z*-*AR* has become a gene involved in sex determination in *R. rugosa* possessing the ZZ/ZW sex-determining system. To the best of our knowledge, this is the first report showing that *AR*-transgenesis induces female-to-male sex-reversal in a species of vertebrate.

*Dmrt1* and *CYP17* expression is enhanced in the XX sex-reversed gonad as well as in the normally differentiating testis of *R. rugosa* [23], [24]. In mice, *Dmrt1* expression is up-regulated during the late stages of male sex differentiation [26], while Tg over-expression of *Dmrt1* in XX tilapia fish results in partial to complete sex reversal [27]. Our results for *Dmrt1* expression in the ZW ovotestes are compatible with these previous findings. Furthermore, we found that the Type 3 Tg gonad, the masculinized part (the ovotestis) that expressed the exogenous *AR* also expressed *Dmrt1* and *CYP17*, while the non-masuculinized part (the ovary) did not express any of these 3 genes (Fig. 3, lanes 3 & 4). In addition, the type 3 ovary (LG) also expressed the *AR*, but showed a little lower expression of *AR* compared to that of the ovotestis (RG) (Fig. S2). However, it is not clear from the present study that the difference in *AR* expression levels between the RG and LG is statistically significant, since the size of the RG was too small to carry out the necessary analysis. However, these results suggest that the influence of *AR* expression on the process of male sex determination may be dosage-dependent, as perhaps might be expected. However, why exogenous *AR* was expressed in one gonad but not in the other of some Tg ZW frogs requires further investigation.

ZW female frogs can develop testes in the absence of Tg *AR* when T is supplied. In the ZW testis, *Dmrt1* and *CYP17* mRNA levels increase, as they do in the developing ZZ testis, indicating that T regulates *Dmrt1* and *CYP17* expression directly or indirectly through the AR. Threshold levels of T and *AR* in the ZW gonads required for sex determination have yet to be determined; amounts available may be insufficient to trans-activate cognate genes, or to initiate the gene cascade necessary for specification of the male fate. However, a ZW gonad would be expected to mature into testes if the combined levels of T and AR were sufficient for testis formation. In support of this is the finding that more androgens are synthesized in *R. rugosa* Wt ZZ gonads during sex determination than in Wt ZW gonads [17]. To determine what these androgen levels are and what dosage combination of T and AR can induce ovotestes and complete sex-reversal in genetically female frogs, further study is needed on Tg ZW gonads, a challenge made difficult by the size of tadpole gonads during sex determination. Nevertheless, it is clear from the present study that exogenous *Z*-*AR* can induce sex-reversal in ZW *R. rugosa*, suggesting that androgen and its receptor are involved in non-genotypically programmed male sex determination in this species, and moreover, this report provides evidence for the first time that evolutionary degradation of the *W-AR* has led to a critical role for the AR (*Z*-*AR*) in sex determination in a species of vertebrate.

## Supporting Information

Figure S1 **Histology of the type 3 gonads.** All 3 of the Type 3 Tg ZW gonads obtained in this study are shown (a, b and c). Dashed lines indicate the borders of the gonads.
(TIF)

Figure S2 ***AR*** **expression in Wt ZW (+T) gonads.** The PCR analysis was performed to examine *Z-AR* expression in Tg and Wt ZW gonads treated with (+) or without (−) T as described elsewhere [16]. Primers used for PCR analysis of *Z*-*AR* and *GA3DPH* expression are shown in Table 1. The sex of each frog was determined as previously described [16]. Upper and lower panels indicate *Z-AR* and *GA3PDH* mRNA level in the gonad, respectively.
(TIF)

Figure S3 **Immunohistology of Wt ZW (+T) gonads.** Frozen sections from the Wt ZW (+T) testis were stained with the antibodies of AR, CYP17, Vasa and laminin (Lam), and counterstained with DAPI and also HE. AR- and CYP-17 positive signals were observed in the Wt ZW testis treated with T.
(TIF)
