# Supplementary figures and images for: Involvement of Androgen Receptor in Sex Determination in an Amphibian Species
Source: PLoS One. 2014 May 14;9(5):e93655. doi: 10.1371/journal.pone.0093655 (PMC4020753; doi:10.1371/journal.pone.0093655)

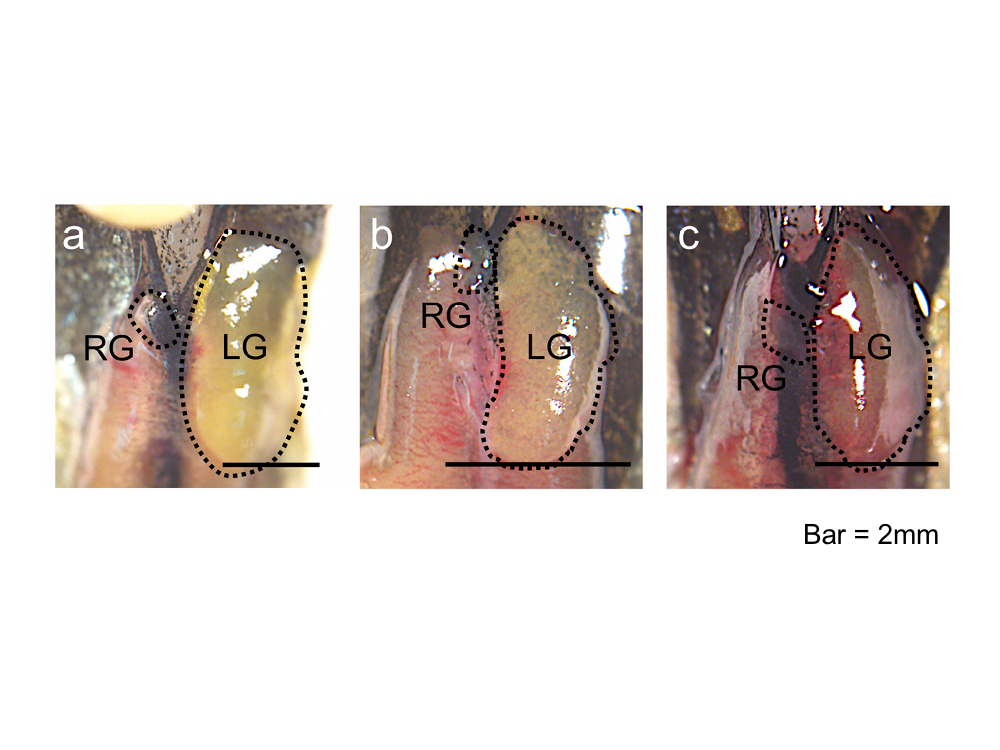

Supplement: Figure S1 — Histology of the type 3 gonads. All 3 of the Type 3 Tg ZW gonads obtained in this study are shown (a, b and c). Dashed lines indicate the borders of the gonads. (TIF) [file pone.0093655.s001.tif]

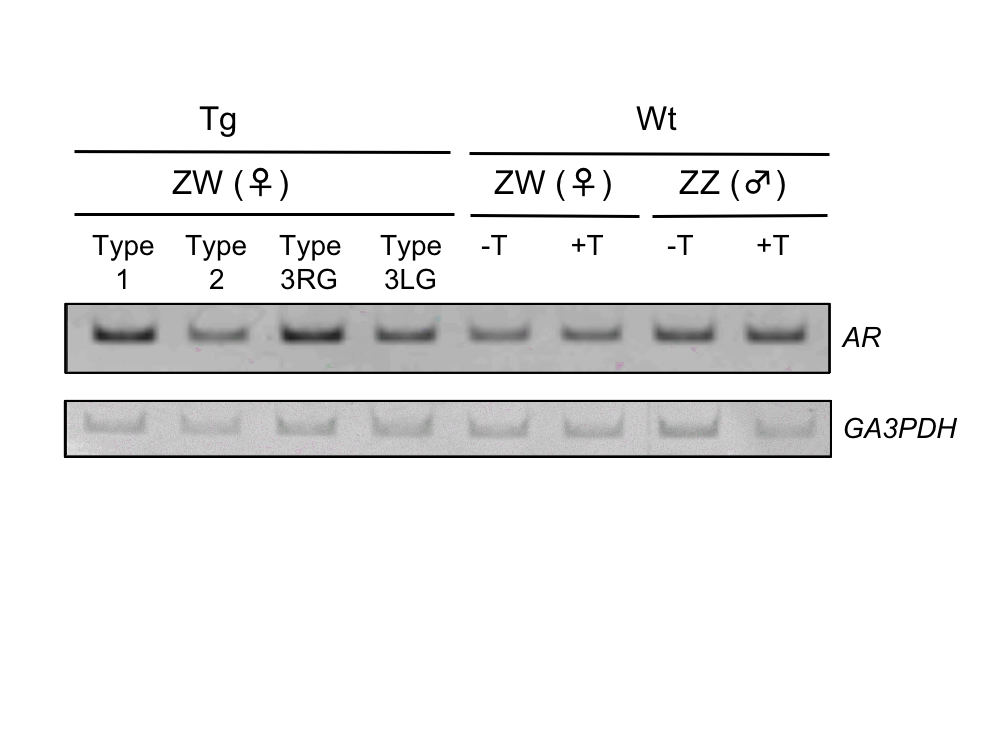

Supplement: Figure S2 — AR expression in Wt ZW (+T) gonads. The PCR analysis was performed to examine Z-AR expression in Tg and Wt ZW gonads treated with (+) or without (−) T as described elsewhere [16]. Primers used for PCR analysis of Z-AR and GA3DPH expression are shown in Table 1. The sex of each frog was determined as previously described [16]. Upper and lower panels indicate Z-AR and GA3PDH mRNA level in the gonad, respectively. (TIF) [file pone.0093655.s002.tif]

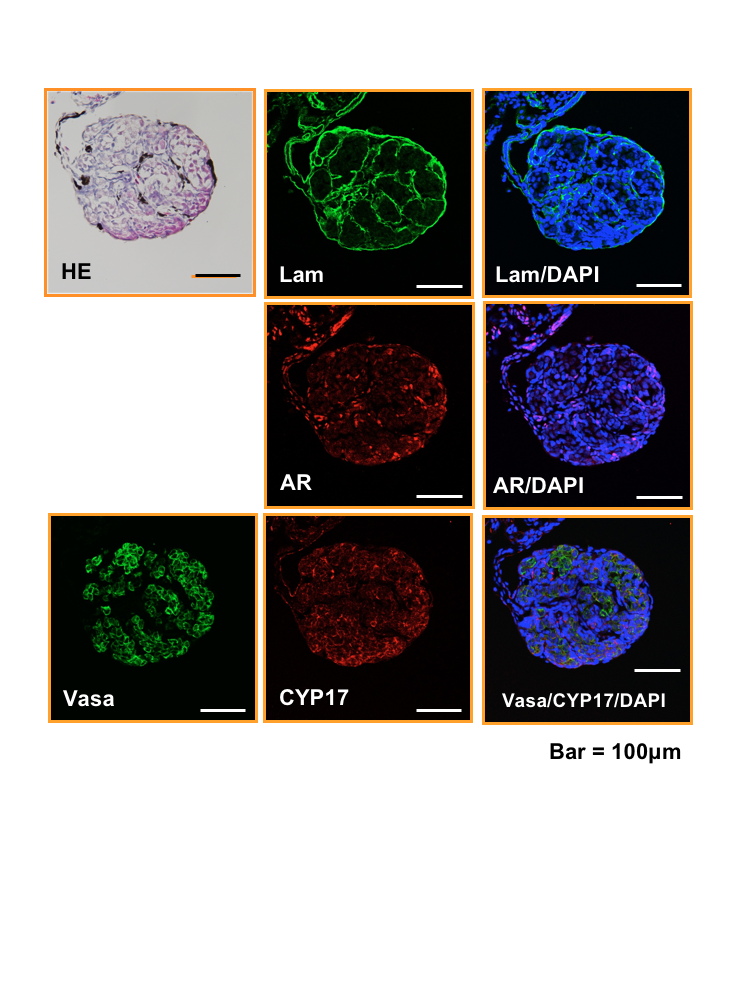

Supplement: Figure S3 — Immunohistology of Wt ZW (+T) gonads. Frozen sections from the Wt ZW (+T) testis were stained with the antibodies of AR, CYP17, Vasa and laminin (Lam), and counterstained with DAPI and also HE. AR- and CYP-17 positive signals were observed in the Wt ZW testis treated with T. (TIF) [file pone.0093655.s003.tif]
